# Mixed-phenotype (B-lymphocytic/myeloid) acute leukemia with ETV6-ABL1 expression

**DOI:** 10.12669/pjms.40.6.8497

**Published:** 2024-07

**Authors:** Luting Wang, Dabao He, Lina Lu, Heng Wang, Suyun Wang

**Affiliations:** 1 Luting Wang Department of Hematology, Shenzhen Longhua District Central Hospital, Shenzhen 518110 Guangdong, China; 2 Dabao He Department of Clinical Laboratory, Shenzhen Longhua District Central Hospital, Shenzhen 518110, Guangdong, China.; 3 Lina Lu Department of Hematology, Shenzhen Longhua District Central Hospital, Shenzhen 518110 Guangdong, China; 4 Heng Wang Department of Hematology, Shenzhen Longhua District Central Hospital, Shenzhen 518110 Guangdong, China; 5 Suyun Wang Department of Hematology, Shenzhen Longhua District Central Hospital, Shenzhen 518110 Guangdong, China

**Keywords:** Mixed-phenotype acute leukemia, ETV6-ABL1, TKI

## Abstract

**Objectives:**

Mixed-phenotype acute leukemia (MPAL) is rare in the clinic, accounting for approximately 2%-5% of acute leukemia cases.

**Methods:**

In this study the cohort included 126 patients, of which 125 cases were from re-examined published data and current patients from Shenzhen Longhua District Central Hospital, carrying an ETV6-ABL1 rearrangement from April 15, 2020 to December 19, 2020. The ETS variant transcription factor 6-Abelson proto-oncogene 1 (ETV6-ABL1) fusion gene is mainly seen in malignant hematological diseases such as acute myeloid leukemia (AML), acute lymphocytic leukemia (ALL), myeloproliferative neoplasms (MPNs). Positivity of both MPAL and ETV6-ABL1 suggest a poor prognosis. This is the first report of B lymphocytic/myeloid mixed-phenotype acute leukemia with ETV6-ABL1 fusion gene positivity. Complete remission was achieved with chemotherapy for lymphoid and myeloid leukemia and targeted therapy with tyrosine kinase inhibitors (TKIs).

**Results:**

The level of ETV6-ABL1/ABL decreased from 23.056% to 11.165%. After consolidation chemotherapy, the patient underwent allogeneic hematopoietic stem cell transplantation but died due to intestinal rejection. There are 126 cases of ETV6-ABL1 fusion gene transcript expression in numerous hematologic malignancies reported to date, including 48 cases of ALL, 12 of AML, and 65 of MPN. Eosinophilia is a common characteristic, especially in MPN patients.

**Conclusion:**

Survival analysis suggests that the survival time of ALL and MPN patients receiving TKI treatment is better than that of patients not receiving this treatment. Dasatinib or nilotinib, especially the former, is more effective than imatinib for MPN.

## INTRODUCTION

Morphology, immunology, cytogenetics, and molecular biology (MICM) are the basis of diagnosis and treatment of acute leukemia. Acute myeloid leukemia (AML), acute B lymphoid leukemia (B-ALL), and acute T lymphoid leukemia (T-ALL) are subtypes of acute leukemia, whereas MPAL is an extremely rare type that has specific immunophenotypic markers from multiple lineages. MPAL is classified according to immunophenotype as B-lymphocytic/myeloid, T-lymphocytic/myeloid, B-lymphocytic/T-lymphocytic, or trilineage combinations. More than half of MPAL cases are the B/myeloid type.^1^ The ETV6-ABL1 fusion was first reported in 1995, in a child with B-cell precursor ALL (BCP-ALL).^2^ To date, 126 cases have been reported, including in 48 cases of ALL, 12 cases of AML, and 65 cases of MPN. ETV6-ABL1, a nonreceptor tyrosine kinase, is similar to the BCR-ABL1 fusion in terms of disease pathogenesis and unfavorable clinical outcome.^3^

However, as the ETV6-ABL1 fusion is rare, its incidence leukemia is lower than that of BCR-ABL1.^4^ ETV6-ABL1 is detected in some types of hematologic malignancies, such as acute leukemias predominating in children and young adults and MPN mainly occurring in older adults.^4^ The ETV6 gene contains two regions homologous to the ETS variant transcription factor, producing two types of oncogenic proteins with increased tyrosine kinase activity via fusion with the ABL1 gene.^5^ Patients who carry and express the fusion typically present initially with myeloid/lymphoid proliferation with eosinophilia/basophilia and show hypersensitivity to TKI treatment.^6^ Here, we present new data for a ETV6-ABL1 patient and thoroughly scrutinize existing data from other patients harboring this fusion to describe clinical features, outcome and efficacy of treatment.

## METHODS

This report, included 126 patients, including 125 cases which were from re-examined published data and current patients from Shenzhen Longhua District Central Hospital, carrying an ETV6-ABL1 rearrangement from April 15, 2020 to December 19, 2020.

### Ethical Approval:

The study was approved by the Institutional Ethics Committee of Shenzhen Longhua District Central Hospital (No.:2020034; date: March 13, 2020), and written informed consent was obtained from all participants.

A 37-year-old male was admitted to Shenzhen Longhua District Central Hospital for muscle pain, leukocytosis, thrombocytosis and erythropenia on April 13, 2020. Complete blood counts revealed white blood cells at 52.3 ×10^9^/L with blasts, red blood cells at 2.67 ×10^12^/L, hemoglobin at 80 g/L, and platelets at 652×10^9^/L. On physical examination, sternal tenderness and palpable splenomegaly measuring 8-cm below the left subcostal margin were noted.

**Fig.1:**
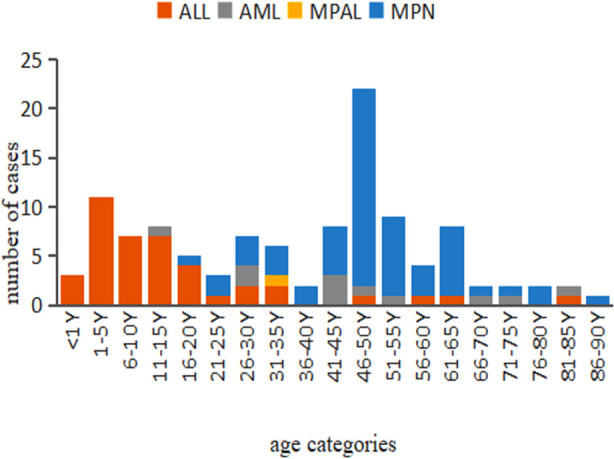
Malignancy subtypes and age of the ETV6-ABL1-positive cases.

A bone marrow(BM) aspirate smear showed active nucleated cell proliferation, with 22.5% myeloblasts and 6% lymphocytes; the latter, among which primitive lymphocytes were seen, were mainly mature lymphocytes. Cytochemical staining indicated myeloperoxidase (MPO) positivity. A diagnosis of acute myeloid leukemia was considered based on the appearance of the bone marrow (Fig.1). On April 15, 2020, the patient was treated with the DA regimen (daunorubicin 150 mg days 1-3+cytarabine 80 mg days 1-5) and imatinib (600 mg/d). Because of the patient’s “dry tap”, a diagnostic bone marrow aspirate was unobtainable. Flow cytometry analysis of peripheral blood cells showed that the myeloid progenitor cell population accounted for approximately 1.1% of nucleated cells, expressing CD34+, CD117+, HLADR+, CD33+, CD13+, CD7+, and CD19-. In addition, primitive/naive B lymphocytes accounted for approximately 1.0% of all nucleated cells and expressed CD19+, CD10+, CD34+, HLADR+, CD33+, CD117-, and CD20-, CD7-, CD38-, CD58 +, CD22+, and CD200+. A bone marrow biopsy specimen revealed B-lineage acute lymphoblastic leukemia. The immunophenotype was characterized by expression of CD34, TdT, CD20, PAX-5 and CD10 but a lack of CD117, MPO and CD3 expression. PQ-PCR analysis showed positivity for the peripheral blood ETV6-ABL1 fusion transcript, with ETV6-ABL1/ABL quantitative results of 23.056%.

As mentioned above, over 20% blasts were observed on the bone marrow aspirates smear, which is consistent with the diagnosis of acute leukemia. The bone marrow smear was also positive for peroxidase staining, though the neutrophil alkaline phosphatase score was very low. According to the bone marrow smear results, we considered the possibility of myeloid leukemia. DA regimen chemotherapy was given on April 15, 2020. Subsequently, the bone marrow tissue was characterized and found to be infiltrated by blasts, which were positive for CD34, TdT, CD20, PAX-5 and CD10. Flow cytometry indicated an inactive B-lymphocytic/myeloid phenotype. Combined with the abnormal fusion gene results, we made a diagnosis of mixed-phenotype (B-lymphocytic/myeloid) acute leukemia with high-risk ETV6-ABL1 expression. Allogeneic hematopoietic stem cell transplantation (HSCT) is necessary for patients after complete remission, and we changed the chemotherapy to that for lymphoid and myeloid leukemia and targeted therapy with tyrosine kinase inhibitors (TKIs). VP regimen chemotherapy, prednisone acetate at a dose of 75-mg daily and vindesine at four mg on days one, eight, fifteen, and twenty were administered beginning on April 15, 2020, and imatinib at a dose of 600 mg daily was added.

On May 7, 2020, re-examination of bone marrow smears showed a roughly normal proportion of granulocytes, of which primordial granulocytes accounted for 1% and promyelocytic granulocytes for 2.5%. In addition, the smear showed that lymphocytes accounted for 32%, with primitive lymphocytes accounting for 4%. Approximately 0.2% of primitive/naive B lymphocytes and 0.1% of myeloid blasts were observed in the patient’s bone marrow flow cytometry specimens. The quantitative ETV6-ABL1/ABL ratio based on PQ-PCR analysis of the bone marrow blood was 11.165%. Complete remission was considered to have been achieved with induction chemotherapy. On May 18, 2020, re-examination of bone marrow smears revealed a primitive lymphocyte proportion of 1%, and approximately 0.02% of primitive/naive B lymphocytes and 1.1% of myeloid blasts were observed in bone marrow flow cytometry specimens. PQ-PCR analysis showed a quantitative TEL-ABL1/ABL ratio of 12.567% for bone marrow blood. On May 20, 2020, the patient received consolidation chemotherapy with DA+VP+imatinib.

On July 31, 2020, bone puncture in another hospital indicated complete remission, and a second course of consolidation chemotherapy was performed. After the second course of consolidation chemotherapy, allogeneic hematopoietic stem cell transplantation will be performed, and the bone marrow transplantation warehouse will be released on October 13, 2020. The patient died of intestinal rejection after transplantation on December 19, 2020. The overall survival period was eight months.

### Bone marrow cell morphology:

Using conventional Wright staining, 200 cells were analyzed on bone marrow smears, and 100 white blood cells were analyzed on blood smears. Bone marrow smears were processed for peroxidase staining (POX), nonspecific esterase staining (NSE), glycogen staining (PAS) and other staining.

### Flow cytometry:

Immunophenotyping was performed by flow cytometry using monoclonal antibodies against CD5(2), CD7, CD56(2), CD8, CD4, CD3, CD2, CD10, CD19(2), CD20, CD14, CD13, CD64, CD16, CD11b, CD15, CD36, CD33, CD34 (2), CD117 (2), CD71, HLADR, CD38, CD138, CD200, CD61, CD45(6), sIg-Kappa, and sIg-Lambda.

### Quantification of ETV6-ABL1 fusion gene transcripts by real-time fluorescent quantitative PCR:

Real-time fluorescent quantitative PCR was performed to quantify ETV6-ABL1 fusion gene transcripts and the internal reference ABL1 gene. RNA was extracted from blood, and the specimens were sent to Guangzhou Jinyu Medical Laboratory Group Co., Ltd., as the ETV6-ABL1 fusion gene type could not be distinguished using a kit. ETV6-ABL1 gene rearrangement was tested in batches, and single sample data are not exported separately.

### Chromosome analysis:

Chromosome analysis was performed using standard cytogenetic techniques on peripheral blood samples from our patient. The G-banded karyotype of peripheral blood cells showed no analyzable division phases.

Induction and consolidation chemotherapy regimens address the myeloid and lymphatic system, including DA+VP+imatinib, daunorubicin 150 mg/d×3 d, cytarabine 80 mg/d×7 days, vindesine 4- mg/d on day one, eight , 15, and 22, prednisone acetate 75 mg/d ×14~28 daily, and imatinib 600 mg/d. Consolidation chemotherapy or allogeneic hematopoietic stem cell transplantation (allo-HSCT) is carried out after hematological complete remission (CR) is achieved.

### Statistical analysis:

SPSS 26.0 was used for statistical processing. Overall survival (OS) was estimated with the Kaplan-Meier method and compared using a log-rank test. Independent sample t test is used in this study. The sample size is estimated by 95% confidence interval. Statistical significance was defined as *p* <0.05.

## RESULTS

To date, there are 126 cases of ETV6-ABL1 fusion gene rearrangement in numerous hematologic malignancies, including 48 cases of ALL, 12 cases of AML, and 65 cases of MPN (including CML). Detailed information on these 126 patients can be found in the Supplementary Appendix. Among 41 ALL patients whose age was reported, 28 cases occurred in children, and 48 patients who were diagnosed with MPN were over 40 years old (Fig.1). In ALL patients, the ETV6-ABL1 fusion gene is mainly seen in children, whereas ETV6-ABL1 positivity in MPN is mainly found in middle-aged and elderly patients.

In cases for which white blood cell counts have been reported, white blood cell counts over 50×10^9^/L were observed in 16/28 children and 3/13 adults with ALL and nearly half of AML patients. However, 16 of 65 cases with MPN, less than half of patients, had white blood cell counts over 50×10^9^/L. This present case of MPAL also showed a white blood cell count over 50×10^9^/L.

**Fig.2:**
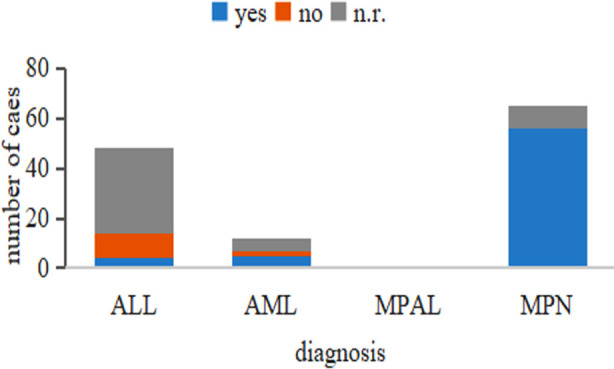
Malignancy subtypes and eosinophilia of ETV6-ABL1-positive cases.

Eosinophilia was observed in more than 66 of the 126 patients with the ETV6-ABL1 fusion and was present in 48 of those whose eosinophil levels were not mentioned. In the 66 patients with eosinophilia, the case distribution was as follows: all MPN cases; five of seven AML cases; the only MPAL case; four of 14 ALL cases. In general, almost all patients with MPN have eosinophilia. In addition, a nearly 2:1 male predominance was observed, and ages ranged from eight months to 88 years (Fig.2).

**Fig.3:**
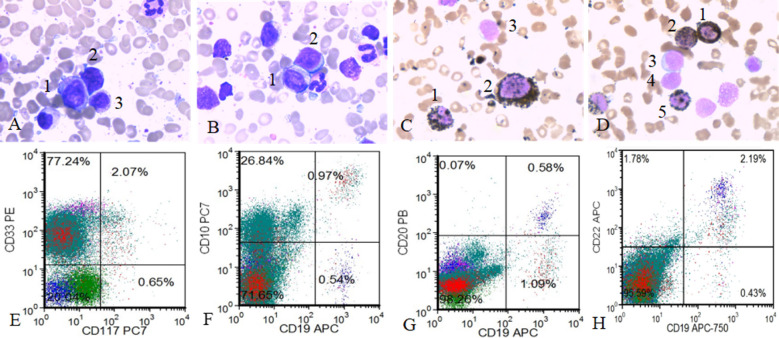
Bone marrow cell morphology and flow cytometry results. A 1. myeloblast 2. lymphoblast 3. immature granulocyte; B 1. myeloblast 2. lymphoblast; C 1. MPO+ mature granulocyte 2. MPO+ blast 3. MPO- lymphoblast; D 1-2 MPO+ immature granulocyte 3. MPO- lymphoblast 4. MPO- prolymphocyte 5. MPO+ immature granulocyte; E-H flow cytometry CD33+, D117+, CD19-, CD10+, CD22+, CD20- immunophenotype

The bone marrow (BM) aspirate smear revealed active nucleated cell proliferation, with 22.5% myeloblasts and 6% lymphocytes. Lymphocytes, among which primitive lymphocytes were seen, were mainly mature lymphocytes. Cytochemical staining suggested that myeloperoxidase (MPO) was partially positive. According to flow cytometry analysis of peripheral blood cells, the myeloid progenitor cell population accounted for approximately 1.1% of nucleated cells expressing CD34+, CD117+, HLADR+, CD33+, CD13+, and CD7+ but CD19-. In addition, primitive/naive B lymphocytes accounted for approximately 1.0% of all nucleated cells, expressing CD19+, CD10+, CD34+, HLADR+, CD33+, CD117-, CD20-, CD7-, CD38-, CD58+, CD22+, and CD200+ (Fig.3). Bone marrow biopsy specimen indicated B-lineage acute lymphoblastic leukemia. The immunophenotype was characterized by expression of CD34, TdT, CD20, PAX-5 and CD10 but not of CD117, MPO or CD3.

### Survival analysis:

**Fig.4:**
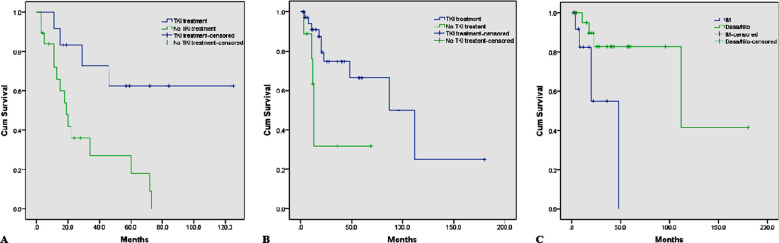
Overall survival of ETV6-ABL1-positive patients receiving different therapies. A: Survival analysis between TKI treatment and no TKI treatment in ALL patients. B: Survival analysis between TKI treatment and no TKI treatment in MPN patients. C: Survival analysis between imatinib and dasatinib/nilotinib of MPN patients.

Based on the current statistics from reported cases with treatment and outcome data, we conducted an overall survival analysis for ALL and MPN cases (Fig.4). A survival difference was noted between ALL patients who did and did not undergo TKI therapy (*p*= 0.004). The median OS of ALL patients treated with TKIs was not estimated because the outcome of most cases reported in the literature was not death, resulting in a censored percentage of 66.7%. In contrast, the OS of ALL patients not treated with TKIs(n=19) was 19 months, ranging from 12-25 months. For MPN cases, survival curve analysis demonstrated higher overall survival for the TKI treatment group than the non-TKI treatment group (median 87 months, range 37-136 months, vs. median 13 months, range 11-14 months; *p*=0.01). We have taken permission to use the photographs in the write up.

It should be noted that one CML patient with a survival period of 240 months was not included in the survival analysis. This case, only treated with interferon-α, differs from previously reported cases by its indolent course.^7^ Thirteen patients (33%) were initially treated with imatinib, whereas 23 patients were initially treated with dasatinib or nilotinib and subsequently received a second-generation TKI. Patients with MPN given dasatinib or nilotinib had a better OS than patients only receiving imatinib (median 48 months, vs. median 112 months; *p*= 0.08).

## DISCUSSION

To date, there have been 126 cases of the ETV6-ABL1 fusion gene in numerous hematologic malignancies, including 48 cases of ALL, 12 cases of AML, and 65 cases of MPN (including CML). Patients with MPN account for the largest proportion, followed by ALL. In ALL, ETV6-ABL1 is mainly seen in children; in MPN, TEL-ABL1 is mainly seen in middle-aged and elderly patients. ETV6-ABL1 rearrangements have been reported for the first time in patients with MPAL, with value and supplementing the disease spectrum.

Mixed-phenotype acute leukemia(MPAL) is rare in the clinic, accounting for approximately 2–5% of acute leukemia cases^8^, with features of positivity for more than two of three expression markers in the myeloid lineage, B lineage and T lineage.^9^ MPAL patients have poorer survival than similar-aged patients with ALL.^10^ Two approaches of classifying MPAL are based on the European Group for the Immunological Characterization of Leukemias(EGIL) and the relevant standards revised by the World Health Organization(WHO) in 2016. Cytochemical staining of samples from our patient suggested that myeloperoxidase was positive. Flow cytometry analysis showed an immunophenotype involving expression of CD13, CD33, CD117, CD19, and CD22. The immunophenotype of bone marrow biopsy is characterized by expression of CD34, TdT, CD20 and CD10.

According to the EGIL scoring system, both a myeloid lineage and a B-lymphoid lineage are assigned greater than two points, suggesting the diagnosis of mixed B/myeloid acute leukemia. The 2016 revision to the WHO classification, which did not change the diagnosis of specific markers compared with the 2008 revision^11^, also supports this patient’s diagnosis of MPAL. Knowledge of MPAL with recurrent cytogenetic abnormalities is limited. The t(9;22)(q34;q11.2)/BCR-ABL rearrangement is most common, approximately in 15% of cases.^12^

ETV6-ABL1 rearrangements, also known as TEL-ABL1, have been published as case reports for ALL, AML, and MPN.^6^ In general, acute leukemia with ETV6-ABL1 positivity indicates poor prognosis and prenatal origin. However, the prognostic significance of ETV6-ABL1 chronic myeloproliferative neoplasms is unclear due to the lack of clinical cases and controlled clinical trials. The reason why the ETV6-ABL1 gene is rare is because reverse positioning of the ETV6 and ABL1 genes requires at least three DNA breaks.^13^ Moreover, cryptic translocation between chromosomes nine and 12 is difficult to detect by conventional cytogenetics.^14^ Some scholars conclude that cytogenetic analysis is an important tool for the identification of ETV6-ABL1 fusion genes.

Additionally, FISH and RT-PCR are recommended for early identification.^15^ Compared with conventional techniques, RNA sequencing is more suitable as a diagnostic method because it can identify partners involved in gene fusions and the exact gene sequences for monitoring purposes.^16^ To further our understanding about the ETV6-ABL1 fusion and promote detection, many teams are using large-scale samples to screen for this fusion gene, as described in the Supplementary Appendix. Certainly, the increase in the number of patients suggests that the true incidence rate may be higher.

Eosinophilia is a common characteristic^17^ observed in nearly half of patients. In 66 patients with reported eosinophilia, the number of cases included all MPN cases, half of AML cases, and the only MPAL cases but only four of 14 ALL cases. In addition, there is a nearly 2:1 male predominance, and age ranges from eight months to 88 years. Therefore, we considered that almost all patients with MPN and half of patients with AML have eosinophilia but no ALL patients. Thus, it remains unclear whether MPAL patients develop eosinophilia. Morphological features are more common in male patients and appear in a wide range of ages. In patients with MPN, eosinophilia can be a ubiquitous feature.

There are A or B types of ETV6-ABL1 transcripts, all formed by the ABL1 gene at 9q34 and the ETV6 gene at 12p13. The difference between these two transcripts is that the type B variant contains TEL exon-5.^4^ The ETV6-ABL1 oncoprotein and BCR-ABL1 protein can both lead to activation of the nonreceptor tyrosine kinase ABL1, which stimulates similar downstream pathways affecting cellular survival, growth rate, independence and transforming capacity.^18^ Because of the similar molecular pathogenesis of driven leukemogenesis between BCR-ABL1 and ETV6-ABL1, tyrosine kinase inhibitors have been considered to be effective in some patients carrying the ETV6-ABL1 fusion.^6^

However, TKI resistance is problematic in TKI-treated patients, and the molecular mechanisms are probably due to the T315I mutation, K89M mutation and ETV6-ABL1-independent TKI resistance in a single patient.^19^ For AML patients, 6 of 12 were given TKIs, but the longest survival time was seven months. Our case of MPAL, which was treated with DA+VP+imatinib, may be considered to have been effectively treated with a TKI based on changes in the level of the ETV6-ABL1 fusion.

### Limitations:

Because our data were all from case reports and initial events are the diagnosis of the disease, the cutoff of the survival time observation period for many cases was not a death event, which is called right censoring. Furthermore, the survival time was longer in the TKI treatment group than in the other treatment groups, and our survival analysis can only reflect differences to a certain extent. The results confirm that TKI treatment is effective for ALL and MPN patients. In MPN patients treated with TKIs, even those who subsequently received a second-generation TKI due to progressive or relapsed disease had a better OS than patients who only received imatinib. Individual cases support dasatinib as the preferred therapy for atypical CML with ETV6- ABL1.^20^

## CONCLUSIONS

Overall, the outcome of ETV6-ABL1 fusion is often poor, but ETV6-ABL1-positive patients have an excellent treatment response. We recommend using TKI treatment, especially second-generation TKIs such as dasatinib or nilotinib.

### Authors’ Contributions:

**LW** and **SW:** Carried out the studies, participated in collecting data, and drafted the manuscript, are responsible and accountable for the accuracy and integrity of the work.

**DH** and **LL:** Performed the statistical analysis and participated in its design.

**HW:** Participated in acquisition, analysis, or interpretation of data and draft the manuscript.

All authors read and approved the final manuscript.
